# Supplementary material for: Potential Blood Biomarkers for Diagnosing Periprosthetic Joint Infection: A Single-Center, Retrospective Study
Source: Antibiotics (Basel). 2022 Apr 11;11(4):505. doi: 10.3390/antibiotics11040505 (PMC9030667; doi:10.3390/antibiotics11040505)
Supplement: Supplementary file 1 [file antibiotics-11-00505-s001.zip › antibiotics-1629538-supplementary.pdf]

Supplementary Materials:

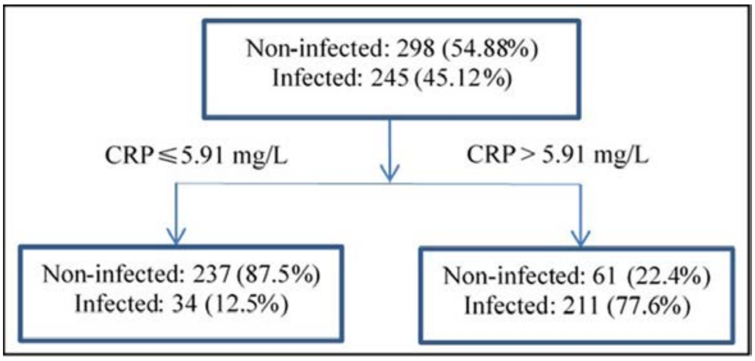

Figure S1. The classification tree of tested markers at a tree depth of one. CRP was enrolled in the classification tree with a cutoff of 5.91mg/L if the tree depth was set as one.

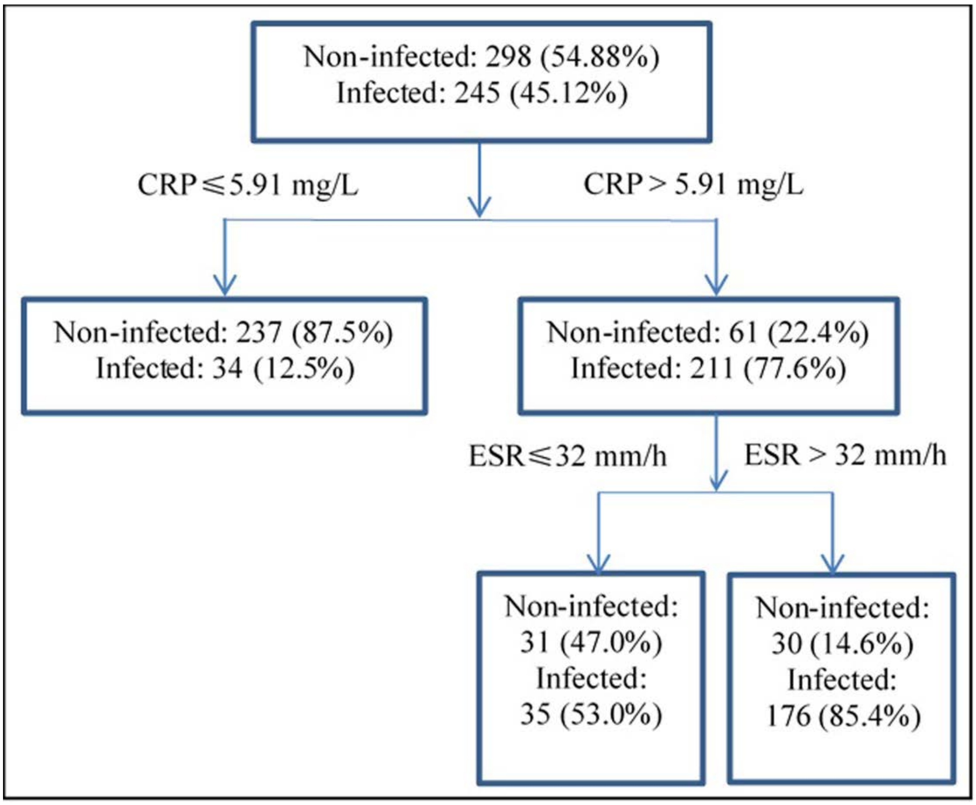

Figure S2. The classification tree of tested markers at a tree depth of two. CRP with a cutoff of 5.91mg/L and ESR with a cutoff of 32 mm/h were enrolled if the tree depth was set as two.

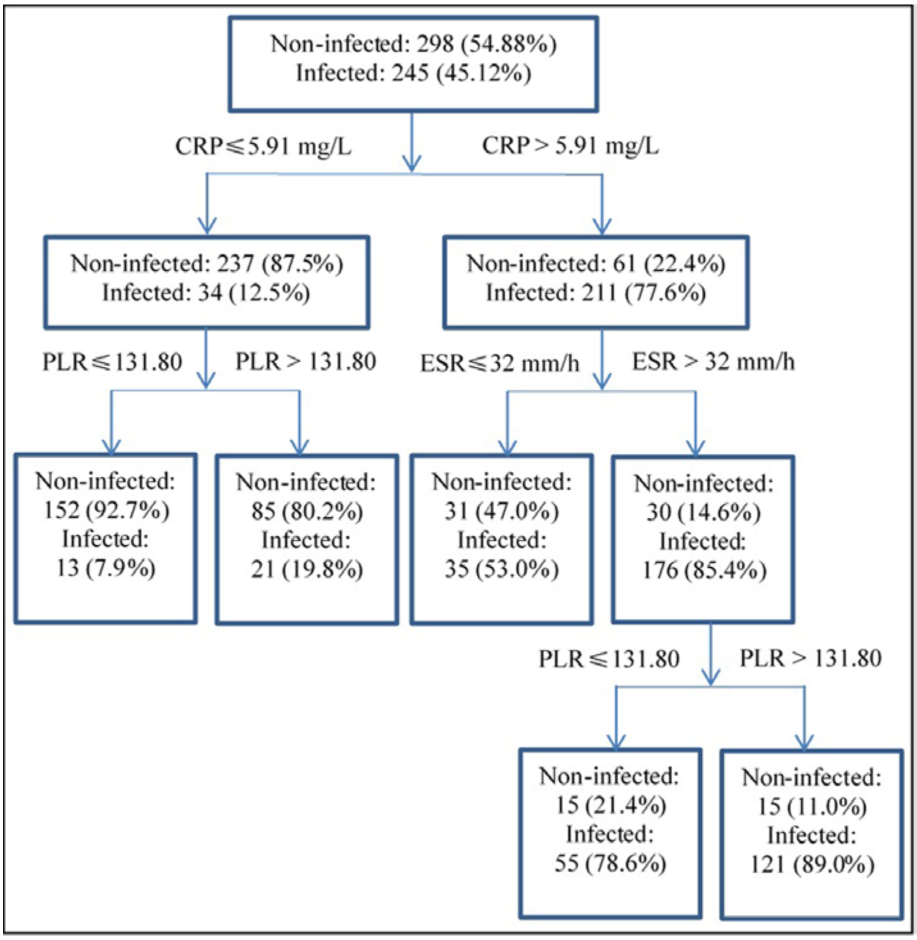

Figure S3. The classification tree of tested markers at a tree depth of three. PLR with a cutoff of 131.80 was successively enrolled following CRP and ESR if the tree depth was set as three.

Table S1. Culture results of the infected patients.

**Variables**	**N composition Ratio (%)**
Culture negative	74 (30.20)
Culture positive	171 (69.80)
Staphylococcus epidermidis	60 (35.09)
Staphylococcus aureus	36 (21.05)
Mycobacterium tuberculosis	8 (4.68)
Pseudomonas aeruginosa	7 (4.09)
Enterobacter cloacae	6 (3.51)
Enterococcus faecalis	5 (2.92)
Escherichia coli	5 (2.92)
Staphylococcus hominis	4 (2.34)
Staphylococcus capitis	4 (2.34)
Hemolytic staphylococcus	3 (1.75)
Staphylococcus warneri	3 (1.75)
Streptococcus intermadius	3 (1.75)
Others	27 (15.77)

Table S2. Diagnostic performance of the two tested markers in combination.

Combination	AUC (95%CI)	Youden Index	Sensitivity	Specificity	PPV	NPV	*P*-value Compared with CRP
CRP combined with other biomarkers							
CRP+ESR	0.873 (0.835-0.910)	0.606	76.4%	84.2%	79.9%	81.3%	0.887
CRP+FIB	0.877 (0.840-0.914)	0.620	75.0%	87.0%	82.6%	80.9%	0.845
CRP+IL-6	0.886 (0.851-0.921)	0.655	80.4%	85.1%	81.6%	84.1%	0.115
CRP+PLT	0.877 (0.841-0.913)	0.630	77.0%	86.0%	81.9%	82.0%	0.643
CRP+MLR	0.887 (0.852-0.922)	0.643	76.4%	87.9%	83.9%	81.9%	0.519
CRP+NLR	0.884 (0.849-0.920)	0.651	79.1%	86.0%	82.3%	83.4%	0.275
CRP+PLR	0.885 (0.850-0.920)	0.644	77.0%	87.4%	83.4%	82.2%	0.256
ESR combined with other biomarkers							
ESR+FIB	0.849 (0.807-0.891)	0.570	68.2%	88.8%	83.4%	77.3%	0.012*
ESR+IL-6	0.852 (0.812-0.892)	0.566	74.3%	82.3%	77.5%	79.6%	0.078
ESR+ PLT	0.818 (0.773-0.863)	0.489	70.3%	78.6%	73.0%	76.3%	0.003*
ESR+MLR	0.822 (0.778-0.862)	0.494	70.3%	79.1%	73.4%	76.4%	<0.001*
ESR+NLR	0.818 (0.773-0.862)	0.494	68.9%	80.5%	74.4%	75.9%	<0.001*
ESR+PLR	0.815 (0.769-0.860)	0.484	64.2%	84.2%	77.0%	74.1%	<0.001*
FIB combined with other biomarkers							
FIB+IL-6	0.867 (0.830-0.905)	0.592	73.2%	86.0%	81.1%	79.6%	0.351
FIB+PLT	0.843 (0.801-0.885)	0.565	71.8%	84.7%	79.4%	78.5%	0.003*
FIB+MLR	0.839 (0.797-0.882)	0.685	68.5%	87.0%	81.3%	77.1%	<0.001*
FIB+NLR	0.836 (0.792-0.879)	0.565	70.5%	86.0%	80.6%	78.0%	<0.001*
FIB+PLR	0.836 (0.793-0.879)	0.571	66.4%	90.7%	85.4%	76.7%	<0.001*
IL-6 combined with other biomarkers							
IL-6+PLT	0.852 (0.813-0.891)	0.592	79.2%	80.0%	76.5%	82.4%	0.104
IL-6+MLR	0.853 (0.815-0.892)	0.584	81.2%	77.2%	74.5%	83.3%	0.094
IL-6+NLR	0.852 (0.813-0.891)	0.575	85.6%	71.6%	71.3%	85.8%	0.075
IL-6+PLR	0.856 (0.817-0.894)	0.590	77.9%	79.1%	75.4%	81.3%	0.143
PLT combined with other biomarkers							
PLT+MLR	0.759 (0.718-0.800)	0.419	59.0%	89.2%	81.8%	72.6%	<0.001*
PLT+NLR	0.753 (0.712-0.795)	0.413	61.1%	80.2%	71.7%	71.5%	<0.001*
PLT+PLR	0.732 (0.689-0.775)	0.392	66.0%	73.2%	66.9%	72.4%	<0.001*
MLR combined with NLR and PLR							
MLR+NLR	0.669 (0.624-0.716)	0.279	48.4%	79.5%	66.6%	65.2%	<0.001*
MLR+PLR	0.707 (0.663-0.750)	0.327	73.0%	59.7%	59.8%	72.9%	<0.001*
NLR combined with PLR							
NLR+PLR	0.700 (0.656-0.744)	0.320	80.7%	48.7%	56.4%	75.4%	<0.001*

* $P < 0.05$. AUC: area under the receiver operating characteristic curve; 95% CI: 95% confidence interval; CRP: C-reactive protein; ESR: erythrocyte sedimentation rate; FIB: fibrinogen; IL-6: interleukin-6; PLT: platelet count; MLR: monocyte/lymphocyte ratio; NLR: neutrophil/lymphocyte ratio; PLR: platelet count//lymphocyte ratio; PPV: positive predictive value; NPV: negative predictive value.
